# Energy Harvesting Device for Smart Monitoring of MV Overhead Power Lines—Theoretical Concept and Experimental Construction

**DOI:** 10.3390/s23177538

**Published:** 2023-08-30

**Authors:** Jozef Bendík, Matej Cenký, Oliver Hromkovič

**Affiliations:** Faculty of Electrical Engineering and Information Technology, Slovak University of Technology in Bratislava, Ilkovičova 3, 81 219 Bratislava, Slovakia; matej.cenky@stuba.sk (M.C.);

**Keywords:** energy harvesting, overhead lines, electric field, measurement, smart grid

## Abstract

Modern technological advancements have opened avenues for innovative low-energy sources in construction, with electric field energy harvesting (EFEH) from overhead power lines serving as a prime candidate for empowering intelligent monitoring sensors and vital communication networks. This study delves into this concept, presenting a physical model of an energy harvester device. The prototype was meticulously designed, simulated, constructed, and tested, to validate its foundational mathematical model, with implications for future prototyping endeavors. The findings illustrate the potential of harnessing ample power from this device when deployed on medium-voltage (MV) overhead power lines, facilitating the monitoring of electric and meteorological parameters and their seamless communication through the Internet of Things (IoT) network. The study focused on the medium voltage applications of the harvester. Two dielectric materials were tested in the present experiments: air and polyurethane. The measurement results exhibited satisfactory alignment, particularly with the air dielectric. Nevertheless, deviations arose when employing polyurethane rubber as the dielectric, due to impurities and defects within the material. The feasibility of generating the requisite 0.84 mW output power to drive process electronics, sensors, and IoT communications was established. The novelty of this work rests in its comprehensive approach, cementing the theoretical concept through rigorous experimentation, and emphasizing its application in enhancing the efficacy of overhead power line monitoring.

## 1. Introduction

In the pursuit of ensuring the future reliability of power grid operations, the imperative of consistent monitoring and communication across numerous critical systems comes to the fore. Among the prevailing challenges, the availability of a suitable power source for remote sensors, encompassing compact meteorological stations, sag monitoring systems, and other Internet of Things (IoT) communication-based devices, emerges as a central constraint [1,2]. The conventional recourse to mitigate power supply predicaments often involves external backup power sources, commonly in the form of batteries, occasionally augmented by supplementary technologies like photovoltaic panels [3]. The present study pivots towards the domain of medium voltage (MV) and elevated voltage tiers within overhead power lines, wherein the focal point rests on the direct energy harvesting principle predicated on capacitance coupling. This premise leverages electric fields proximate to the phase conductor of the power line, constituting the foundational basis for the energy harvesting device under examination. Notably, the key constraint inherent to this proposed paradigm pertains to the low-energy electronics domain—comprising its cost, design intricacies, and operational functionality. The voltage thresholds characterizing such electronics commonly span a few volts—a stark contrast to the tens or hundreds of kilovolts characterizing power line voltage levels. Consequently, the utilization of voltage conversion becomes imperative, albeit diverging from the conventional employment of the standard voltage divider technique, which proves inapplicable within this context.

Current technology developments have opened up possibilities for real-time monitoring of overhead power lines. This monitoring can be used for the measurement of:

- electrical quantities, e.g., voltage and current at a specific location—potentially useful for fault or ground connection of localization [4];
- meteorological parameters, e.g., wind speed, air humidity, solar irradiation, ambient temperature, etc. This information can be used for dynamic power line rating estimation or fault condition prediction online (severe icing on power line conductors, low ground clearance, conductors galloping) [5,6,7];
- mechanical parameters of the line, e.g., sag, conductor vibration [8,9].

A power supply for sensors, data processing, and communication is needed, to achieve practical and reliable monitoring. The following energy sources for energy harvesting have the potential to be used for power line monitoring supply:

- solar energy;
- thermoelectric generation;
- kinetic energy;
- magnetic field energy harvesting (MFEH);
- electric field energy harvesting (EFEH).

Further theoretical analysis of these particular forms of energy and their harvesting potential can be found in [1,6,10]. Complex research on energy harvesting from MV electric fields can be found in J. C. R. Guerra’s dissertation work [11]. A. Harb, in his paper, summarized energy harvesting techniques, power management, power conversion, and battery charging methods [12]. Extensive MFEH and EFEH methods are briefly explained below.

### 1.1. MFEH—Magnetic Field Energy Harvesting

This method is the most commonly used, in practice. However, it is primarily suitable for continuous and loaded lines, since the amount of harvested energy depends on the current flowing through the phase conductor, the magnetic field of which is coupled to the energy harvesting device via magnetic induction [13]. When the actual line current is below a defined threshold value, the energy harvested from the line is insufficient to power the device, so it goes offline. Therefore, an exemplary operation of the power system is needed. This problem can be overcome by using battery storage and an emergency energy supply. However, this makes the system more expensive to manufacture and operate in the long term, due to the degradation of battery systems [13,14]. A typical example of such a device used in practice is a dynamic line rating OTLM sensor. OTLM is a high-end monitoring sensor that measures conductor inclination, phase current, vibrations, etc. The limitation of this device is its minimal operational phase current, 65 A [15].

### 1.2. EFEH—Electric Field Energy Harvesting

Using an electric field as the energy source allows the device independence from the power line loading status. Since the harvested energy relies only on the existing electric field around the conductor, the limiting factor is the voltage level of the power line and the capacity between the line and the harvester. When AC voltage is present between the phase conductor and the ground, a displacement current is created, which depends on the dielectric environment’s properties and the electric field’s intensity around the conductor. This displacement current enables the energy harvesting device’s capacitive energy transfer and accumulation.

If we compare possible energy gains from MFEH and EFEH for the same type of overhead power line, the MFEH can provide much more energy output. On the other hand, EFEH is much more reliable in the long term [16]. The only situations in which the EFEH principle fails are during a short circuit event or maintenance (i.e., the power line is offline).

### 1.3. Review of the Current State of EFEH Development, and Our Contribution

Basic theoretical concepts, device construction, and testing of the EFEH system on a single conductor were presented in a work by X. Zhao et al. [17]. The authors presented a system consisting of a cylindrical anode that surrounded the phase conductor (cathode). The authors claimed to have gained 16.3 mW of output power on a 60 kV overhead power line. Their concept was the inspiration for the system shown in this paper. This cylindrical consent is also theoretically analyzed in [18].

R. Moghe in his work from 2013 presented a non-cylindrical EFEF sensor working on a 35 kV system. His experiments resulted in a gain of continuous power of 17 mW [19]. His work was further developed in 2015, when the device was able to gain 35 mW of power [20].

In 2009, M. Zhu et al. developed an EFEH device for possible use in power stations. The device consisted of cylindrical plates placed in an electric field created by a 10 kV source. One of the plates was grounded. The energy gain of the device was 10.6 mJ in one minute [21].

J. C. R. Guerra, in his dissertation work, investigated another concept for EFEH, which was based on using power line insulators. The concept was experimentally demonstrated on MV, and the author claimed to have gained around 17.1 mW of output power [11].

Y. Yang et al., in their work, presented a so-called power line “sensornet”, a concept of distributed power line sensors powered by EFEH. Their main goal was to provide continuous power line monitoring [4].

Development of fully cylindrical EFEH devices that surround the insulated phase conductor on a low-voltage NN conductor was described by Ch. Keun-Su et al. [22]. Another demonstration in the field of low-voltage electric field energy harvesting was presented in the work of O. Menéndez et al. [16].

Harvesting energy from an MV overhead power line using specially constructed insulators was described by J.C. Rodríguez, whose simulation revealed that the potential of such a device is almost 100 mW [23].

Another different approach to the EFEH system was shown in the work of S. Kang et al. [24]. The authors tested the energy harvesting under actual three-phase 765 kV power transmission lines. In this case, the energy harvesting device was placed between the phase conductor and the earth’s surface. The device was built on Zigbee-based sensors. The measured energy gain was 0.17 mW of output power.

Currently, most EFEH devices are focused either on voltage levels higher than MV or on concepts in which one of the electrodes is grounded. By grounding, it is logically possible to achieve significantly greater energy gains. Our approach consists in using a cylindrical collector at a voltage level of 22 kV. Our work focused on the first phase, which is the content of the presented article: the possible energy gain of the collector and the validation of the mathematical model. If it proves possible to obtain a sufficient amount of energy, then the project will continue with the secondary phase, which is the design of the electronic circuit and the communication peripherals and sensors.

## 2. Materials and Methods

The device concept is based on the described EFEH principle, which is more stable and universal in terms of usage, but more complicated in terms of energy management and overall design. The total accumulated energy inside the device over a specified time must be sufficient for sending the message (or sequence of messages), signalizing its state of operation and specific measured quantity value. The following construction and operation requirements are considered to be the most important, in terms of practical application:

- simple installation and maintenance;
- reliable operation (communication);
- resistant to any weather conditions;
- no direct connection to the ground;
- lightweight (as much as possible).

**Figure 1:**
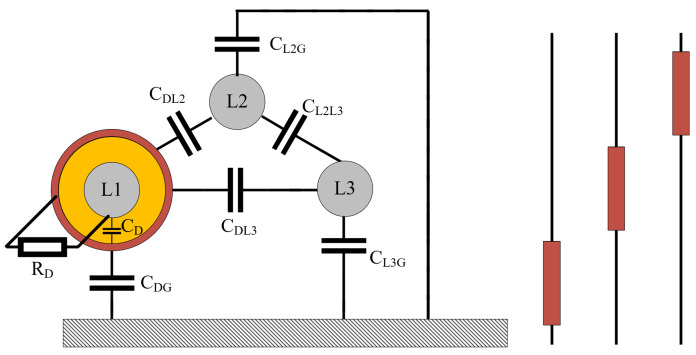
A simplified schematics of the EFEH system (**left**) and the arrangement of multiple devices on a single power line (**right**).

**Figure 2:**
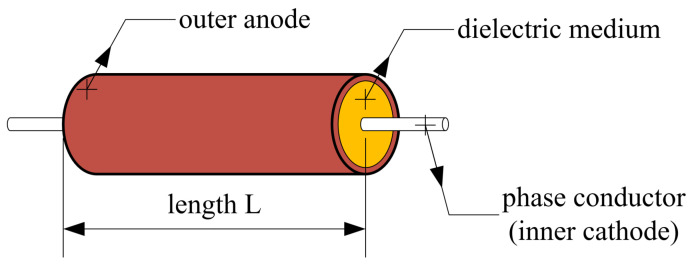
A simplified model of the energy harvesting device.

**Figure 3:**
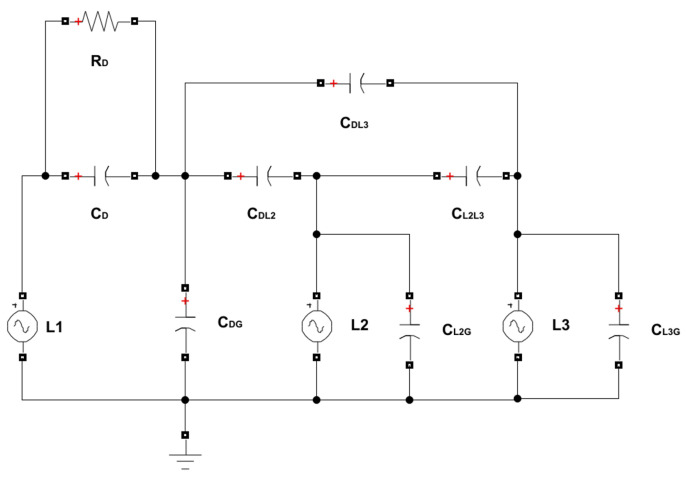
An equivalent scheme of the EFEH system.

In Figure 1, the simplified scheme of the EFEH system can be seen on a general three-phase overhead power line. The resulting design of the device is shown in Figure 2, utilizing the cylindrical shape of the outer electrode wrapped around the phase conductor (inner electrode). This shape enables capacitive coupling between the outer electrode, the ground, and the power line’s other phases. The equivalent scheme of Figure 1 is shown in Figure 3, as modeled in the Matlab Simulink environment. The circuit consists of three phases (voltage sources). Phase *L1* is surrounded by a metal cylinder (EFEH). Parallel with the phases *L2* and *L3* are their capacitances to the ground, $C_{L2G}$, $C_{L1G}$. Capacitance $C_{DG}$ is the capacitance between EFEH and the ground. Capacitance $C_{L1D}$ is the capacitance between Phase *L1* and EFEH. Capacitances $C_{DL2}$, $C_{DL3}$ are the capacitances between EFEH and the other phases. Capacitance $C_{L2L3}$ is the capacitance between phases *L1* and *L2*. Resistance $R_{D}$ connected between phase *L1* and EFEH represents the load (sensor measurement, communication, etc.). The values of the capacitances are calculated using Maxwell potential coefficients [25].

The device can be used in single phase or multiple phases. In the case of multiple phases, the devices need to be shifted from each other by at least their length, L, to work at maximum efficiency and to maintain interphase distances.

**Figure 4:**
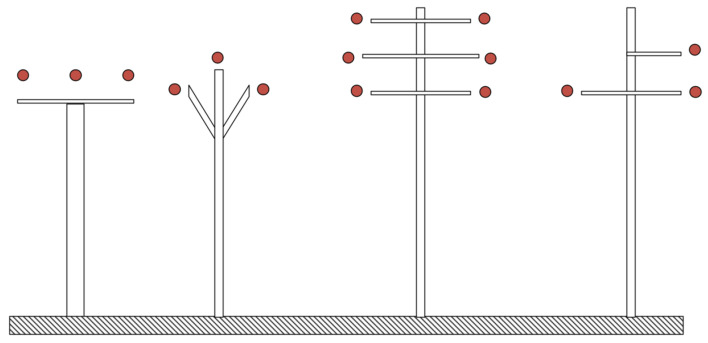
Examples of energy harvesting device placement on MV lines.

The proposed device could be used on any MV overhead power line configuration, as pictured in Figure 4. Due to changes in the geometry of the towers or the influence of new systems, the overall capacitances of all the objects will change. This can affect the overall performance of the EFEH device.

The complete device construction is divided into the following:

- energy harvester part: the cylindrical capacitor around the power line supplies the electronic circuit board inside the device;
- measurement, control, and communication part: everything is contained within the ultra-low-energy circuit board inside the device.

The device can be used for the power line’s diagnostic electrical and non-electrical properties in real time. It is possible to measure the current flowing in the conductor inside the device using the Rogowski coil, to signal the line’s abnormal operational state. As for the non-electric quantities, the proper output can be expected from monitoring:

- conductor temperature;
- environment temperature;
- icing creation;
- solar radiation;
- vibrations of the conductor.

One of the very basic needs of the system is the ability to efficiently and reliably communicate with the rest of the monitoring system infrastructure. In this case, it is also essential to fulfill the condition of the ultra-low-power alternative, because of the character of the power source. For these reasons, IoT technology was chosen as the only communication platform for this device. Available options, in this case, are (in Slovakia), for example, Sigfox SimpleCell, LoRa Orange, or LoRaWAN Slovanet. Each one has different strong and weak points; however, the exact choice is not the point of this paper. The communication requirements are set as follows:

- Regular operation—sending the cumulative measurements every 30 min;
- Faulty/abnormal operation—sending the measurements instantly and the power line status.

**Figure 5:**
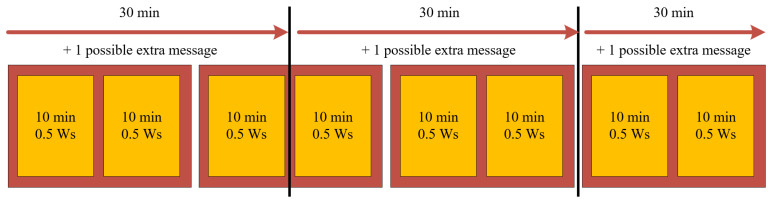
Proposed automatic communication of the device (regular operation).

The measured data should be sent with an adequately set redundancy ratio, increasing the transfer’s reliability at different times. The proposed communication logic is schematically shown in Figure 5. Every 30 min, the device must accumulate enough energy to send four messages, even under the worst conditions (3× regular conditions, 1× abnormal condition). The rough estimation of the communication and electronic board consumption is 0.5 Ws—therefore, at least 0.5 Ws must be accumulated within 10 min if no abnormal condition occurs. To achieve this, 0.84 mW of output power is needed from the harvester. One message is designed as a 10 min window, where the measurements accumulate within the device and are then sent. Single (unique) information is enclosed in a red rectangle, while the single message is in the orange box inside the red rectangle. This system, therefore, fulfills the needs of redundancy and time shifts.

The transmitter uses at least 99% of the total energy consumption, which is a critical component that demands high reliability. Due to limited power management, we decided to choose one-way wireless operation towards the collection point, because the continuous receiving process in a wireless modem uses a significant integral sum of energy that exceeds the short-term energy pulse when sending a message. This consumption cannot be eliminated and is based on circuit design principles. Activating the receiver only at the predicted time is impractical, because it is a non-deterministic event. In addition to signal electronics, the information about the non-receipt of the message to the collection point does not provide relevant information, because the power source cannot cover any failure of the communication network from the energy point of view.

## 3. Measurement and Results

**Figure 6:**
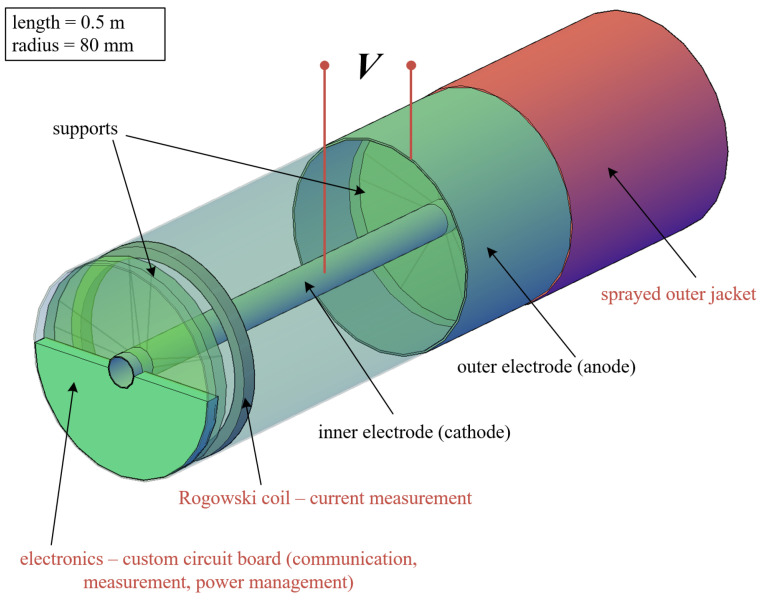
Experimental prototype of the EFEH device—parts whose text is colored in red were not present during the measurement.

As a proof of concept, a prototype of the EFEH device was constructed and tested in laboratory conditions. The specific properties and components of the future planned device are shown in Figure 6.

For the laboratory experiment, a simple device version was constructed. This simpler version did not contain the outer jacket, Rogowski coil, and circuit board with communication. The outer jacket and Rogowski coil were unnecessary for this measurement, while the custom circuit board has yet to be developed, which is the biggest challenge for such a device, as the authors see it. However, this article is meant to verify this specific EFEH concept, not wholly solve the device’s detailed construction. The design of our experiment consisted of:

- development of the mathematical model described in the previous section;
- the construction of a metal (aluminum) cylinder, and a primitive system enabling the attachment and fixation of the cylinder on the 22 kV line;
- the casting of a polyurethane insert, which served as an alternative insulating medium to air;
- the design of the laboratory experiment described in the following section;
- comparison to the mathematical model.

**Figure 7:**
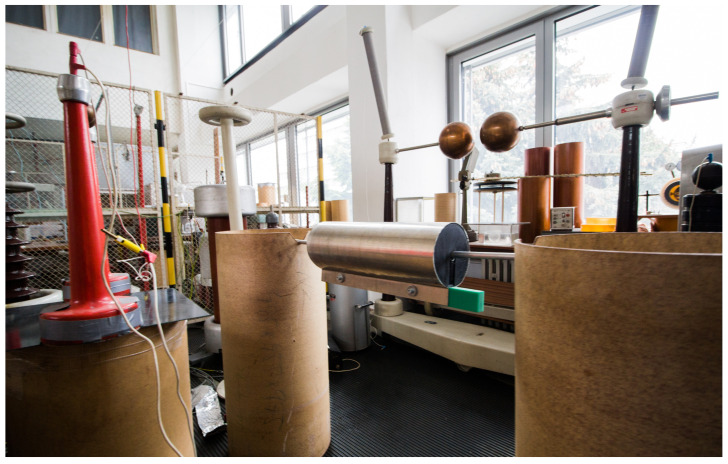
Experimental measurement setup—actual placement in the laboratory conditions.

**Figure 8:**
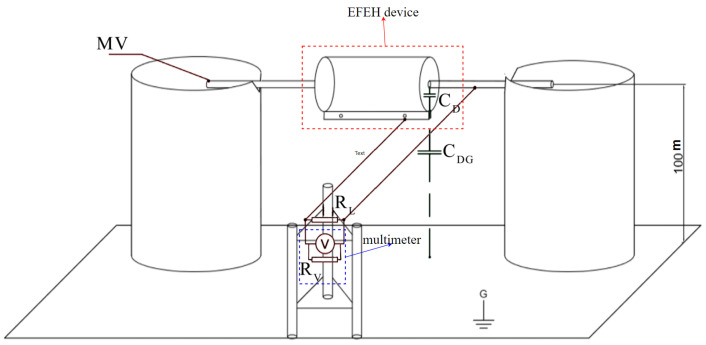
Experimental measurement setup—schematic arrangement tied to the actual placement in the laboratory conditions.

Because of the equipment limitations, the experimental measurement was conducted as a single-phase measurement. The height of the insulation cylinders was 1 m above the ground. The conductor alternative used an aluminium rod connected to an MV power source on the left side (related to Figure 7 and Figure 8). The prototype device had added weight along the cylinder, to ensure its stable position on the aluminium rod.

**Figure 9:**
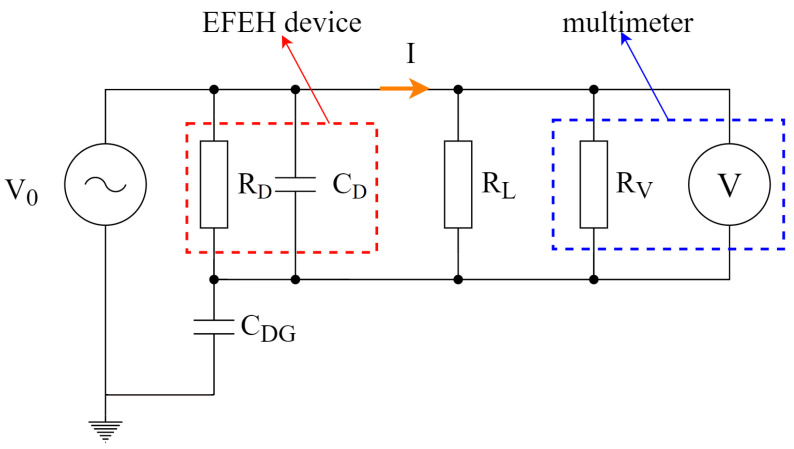
Experimental measurement setup—simplified schematics.

The load used in the experimental setup (noted as $R_{L}$ in Figure 9) was 1.2 MΩ and 8.2 MΩ. The following AC phase-to-ground voltages for each load were applied to the aluminum rod: 4 kV, 8 kV, 12 kV, 16 kV, and 20 kV.

**Figure 10:**
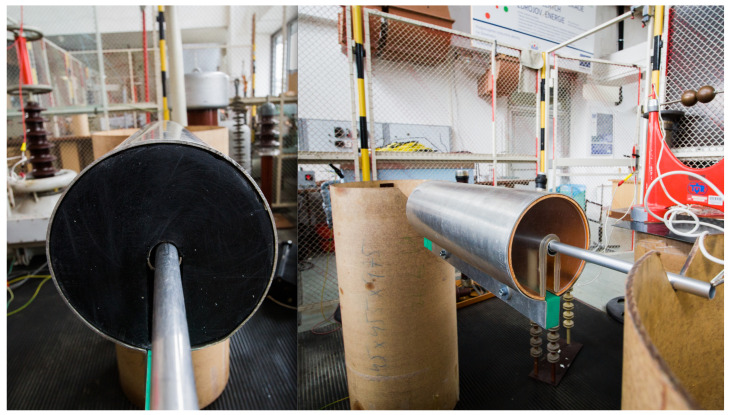
Polyurethane rubber (**left**) and air (**right**)—two dielectric materials were tested.

All the mentioned measurements were carried out for two alternative dielectric mediums in the device:

- polyurethane rubber, relative permittivity $\epsilon_{r}$ = 4, Figure 10 left;
- air, relative permittivity $\epsilon_{r}$ = 1, Figure 10 right.

As shown in Figure 9, the load was connected in parallel between the inner and outer electrodes and the multimeter. The manufacturer defines the multimeter internal impedance ($R_{V}$) as 10 MΩ, which was corrected by our separate measurement, resulting in 10.9 MΩ internal impedance. Also, every $R_{L}$ was separately measured before applying it to the setup. The final impedance was estimated as the parallel combination of the $R_{V}$ and $R_{L}$.

The results are summarized in the following tables. Table 1 shows the result from measurement with polyurethane rubber as insulation, Table 2 shows the result from measurement with air. The most critical parameter was the potentially available power of the EFEH device, which could be sent directly into the circuit board. The legend for the following tables is:

$R_{L}$ load impedance;
*R*   total impedance, parallel sum of $R_{L}$ and $R_{V}$;
$V_{0}$ phase-to-ground conductor voltage;
*V*   voltage between the electrodes;
*I*    output current;
*P*   output power.

## 4. Discussion

**Figure 11:**
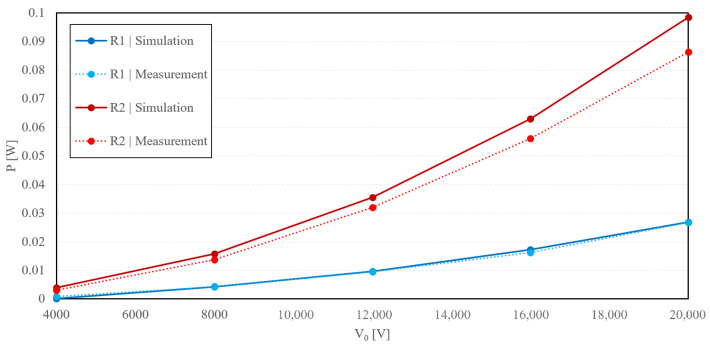
Graphically summarized measured and simulated values for air as the dielectric medium.

**Figure 12:**
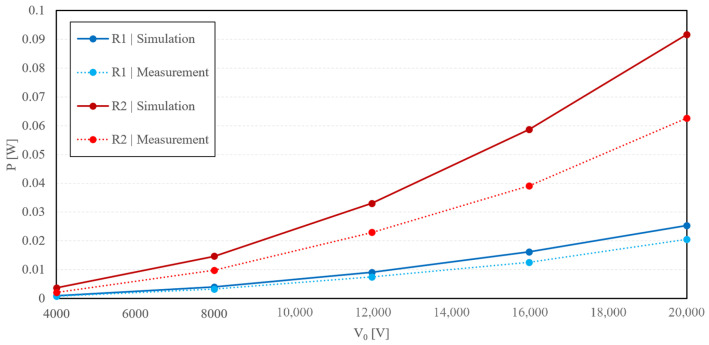
Graphically summarized measured and simulated values for polyurethane rubber as the dielectric medium.

The single-phase simulation was carried out in Matlab Simulink, to verify the measured values, with the scheme shown in Figure 9. As shown in Figure 11 and Figure 12, the simulated and measured output power difference was not over 7% in the case of the air insulator. The polyurethane insulation error exceeded 18%. These discrepancies may have been caused by idealizing the dielectric medium as being completely and homogeneously filled between the electrodes, which was not entirely true. We also suggest that the high error of measurement of polyurethane rubber as the dielectric medium was due to impurities and air bubbles caused by the construction of the device.

After a series of measurements that we performed on the device for both dielectrics, we reached conclusions based on the mathematical model. For all three connected loads in the entire range of applied input voltages to the internal electrode of the collector, higher voltages were achieved, and thus, at the same time, higher performances in the air dielectric.

In the created mathematical model, we considered a simplified design of the device’s inner structure and of the entire structure of the collector.

Thus, the mathematical model did not consider the geometric parts of the clamping mechanism, and, at the same time, in the case of the air model, it did not even consider the enclosing structure. A mathematical model of air uses a dielectric permittivity 1 and a capacity $C_{D}$ of 15.58 pF. A model with a polyurethane insert uses a dielectric permittivity of 4 and a capacity of 60.53 pF. For comparison, we measured its capacity on a real collector, using a Hameg LCR meter, type HM 8118, for both cases of dielectrics. Due to the impossibility of performing measurements at the power frequency, due to device interference, we performed measurements at frequencies close to the power frequency. We calculated the capacitance values for a frequency of 50 Hz from these data, for both cases. For the air model, we thus determined the capacity of 19.9 pF and the collector with PUR material 65.8 pF.

In every case, air as the dielectric medium was more efficient in higher output power for every tested load impedance $R_{L}$ and voltage phase to ground $V_{0}$.

## 5. Conclusions

The proposed prototype of the EFEH device was theoretically designed, simulated, constructed, and measured in laboratory conditions for different phase-to-ground voltages and load impedance. Such a device is meant to operate autonomously on the power line, installed directly on a single conductor. The measurement results indicated satisfactory agreement with the mathematical model, especially for the air dielectric. The measurement using polyurethane rubber as the dielectric showed a higher error caused by PUR impurities and defects. It was estimated that a power supply of 0.84 mW of output power would be needed to power the process electronics, sensors, and communications built by the IoT. The result shows that this is possible; however, the size of RL impedance can still be a problem, and further research and development are needed. Communication of this device was proposed, together with the power supply requirements. This article verified the concept of the EFEH system and showed its possible application in intelligent monitoring systems on overhead power lines.

In conclusion, the presented EFEH device has real potential for practical application. The preferred form of insulation is air, primarily due to its low weight. In the next phase of development, the dimensions and clamping mechanism of the device will be optimized. The development of the electronic part itself, of communication, and of sensors, will follow.

## Figures and Tables

**Table 1:** Results from measurement—polyurethane rubber.

	$R_{L}$ [MΩ]	*R* [MΩ]	$V_{0}$ [kV]	*V* [V]	*I* [µA]	*P* [mW]
R1	1.45	1.28	4	31.10	24.30	0.76
			8	64.50	50.40	3.25
			12	97.50	76.19	7.43
			16	126.60	98.93	12.52
			20	161.90	126.51	20.48
R2	8.23	4.69	4	100.40	21.41	2.15
			8	214.40	45.72	9.80
			12	328.00	69.95	22.94
			16	428.00	91.27	39.06
			20	542.00	115.58	62.65
R3	18.20	6.82	4	137.70	20.20	2.78
			8	309.70	45.43	14.07
			12	450.97	66.15	29.83
			16	601.00	88.16	52.98
			20	724.00	106.20	76.89

**Table 2:** Results from measurement—air.

	$R_{L}$ [MΩ]	*R* [MΩ]	$V_{0}$ [kV]	*V* [V]	*I* [µA]	*P* [mW]
R1	1.45	1.28	4	31.10	24.30	0.76
			8	73.30	57.28	4.20
			12	110.38	86.25	9.52
			16	144.00	112.52	16.20
			20	185.00	144.56	26.74
R2	8.23	4.69	4	119.40	25.46	3.04
			8	253.20	53.99	13.67
			12	387.00	82.53	31.94
			16	513.00	109.40	56.12
			20	636.00	153.63	86.26
R3	18.20	6.82	4	168.80	24.76	4.13
			8	315.60	46.29	14.61
			12	481.00	70.56	33.94
			16	642.00	94.17	60.46

## Abbreviations

The following abbreviations are used in this manuscript:

MFEH	magnetic field energy harvesting
EFEH	electric field energy harvesting
MV	medium voltage
LV	low voltage
